# Persistence time of SIS infections in heterogeneous populations and networks

**DOI:** 10.1007/s00285-018-1222-1

**Published:** 2018-02-23

**Authors:** Damian Clancy

**Affiliations:** Department of Actuarial Mathematics and Statistics, Maxwell Institute for Mathematical Sciences, Heriot-Watt University, Edinburgh, EH14 4AS UK

**Keywords:** Stochastic epidemic models, Large deviations, Endemic fade-out, Directed configuration model, Superspreaders, 92D30, 60J28

## Abstract

For a susceptible–infectious–susceptible infection model in a heterogeneous population, we present simple formulae giving the leading-order asymptotic (large population) behaviour of the mean persistence time, from an endemic state to extinction of infection. Our model may be interpreted as describing an infection spreading through either (1) a population with heterogeneity in individuals’ susceptibility and/or infectiousness; or (2) a heterogeneous directed network. Using our asymptotic formulae, we show that such heterogeneity can only reduce (to leading order) the mean persistence time compared to a corresponding homogeneous population, and that the greater the degree of heterogeneity, the more quickly infection will die out.

## Introduction

In modelling endemic infections, a quantity of particular interest is the persistence time until infection dies out from the population. For discrete state-space Markov chain models, the expected persistence time for an infection that has become endemic in the population (i.e. starting from quasi-stationarity) may be found as an eigenvalue of the transition rate matrix (van Doorn and Pollett 1993). However, for large populations and for more complicated models, numerical computation of this exact solution can be very time-consuming, and may also suffer from numerical instability. Moreover, it is not straightforward to use this eigenvalue characterization to investigate, for instance, the effect of population heterogeneities upon the expected persistence time. Approximation methods are therefore required. For a number of infection models it has been shown (Andersson and Djehiche 1998; Ball et al. 2016; van Herwaarden and Grasman 1995) that, denoting by *N* the typical size of the population, the expected time from endemicity to extinction, $\tau$, is asymptotically given by an expression of the form

1 $$\begin{aligned} \tau \sim {C \over \sqrt{N}} \exp ( AN ) \end{aligned}$$

where the values of *A*, *C* depend upon parameters of the model, but not upon *N*. It is assumed here that the process is super-critical, so that long-term endemicity is possible.

For the classic susceptible–infectious–susceptible (SIS) model of Weiss and Dishon (1971), Andersson and Djehiche (1998) found simple explicit expressions for both *A* and *C* in terms of the basic reproduction number $R_0$ (the expected number of secondary cases caused by a typical primary case in an otherwise susceptible population), under the assumption of super-criticality (that is, $R_0 > 1$); specifically, $A = (1/R_0) - 1 + \ln R_0$ and $C = R_0 \sqrt{2\pi } / ( R_0 - 1 )^2$, assuming time is scaled such that individual infectious periods are of mean 1. This was extended by Ball et al. (2016) to allow for a general infectious period distribution in place of the exponential distribution assumed by Andersson and Djehiche (1998); they showed that leading-order behaviour is unchanged, so that $A = (1/R_0) - 1 + \ln R_0$ as before, while the value of *C* depends upon the infectious period distribution, and may be straightforwardly evaluated provided this distribution is known. Pre-dating the above work, van Herwaarden and Grasman (1995) showed that relationship (1) holds true for a particular susceptible–infectious–removed (SIR) infection model. In this case, however, evaluation of the constant *A* requires numerical solution of a system of ordinary differential equations, while no method for evaluating *C* is given.

The system of ordinary differential equations used in van Herwaarden and Grasman (1995) to evaluate *A* may be regarded as the equations of motion corresponding to a particular Hamiltonian system. More recently, a number of authors (Assaf and Meerson 2010, 2017; Dykman et al. 1994; Elgart and Kamenev 2004; Kamenev and Meerson 2008; Lindley et al. 2014) have applied this Hamiltonian approach to a range of infection models to derive results of the form

2 $$\begin{aligned} \lim _{N \rightarrow \infty } {\ln \tau \over N} = A . \end{aligned}$$

Equation (2) is not as precise as relationship (1), but does at least give the leading-order behaviour of $\tau$ in the large population ($N \rightarrow \infty$) limit. Evaluation of *A* generally requires numerical solution of the equations of motion, and consequently much of the research effort has focused upon developing efficient numerical procedures.

We shall apply the Hamiltonian approach to approximate the expected persistence time from endemicity, $\tau$, for an SIS model incorporating heterogeneity in individual infectivities and susceptibilities. Such heterogeneity is a common feature of real-world infections. For instance, for a number of infections (eg SARS) it has been hypothesised that there exists a subgroup of ‘super-spreaders’ within the population, being individuals of higher infectivity than the rest. Heterogeneous susceptibilities may arise, for instance, through individuals having differing histories of prior exposure to infection or vaccination. Alternatively, our model may be interpreted as a model for infection spreading on an uncorrelated (that is, with no correlations between degrees of neighbouring individuals) directed network (Dorogovtsev et al. 2008).

In contrast to almost all previous work, we are able to find an explicit formula for the constant *A* in Eq. (2), at least provided the heterogeneity is in either infectivity, or susceptibility, but not both. As well as being much quicker and easier to evaluate than the solution to a (typically high-dimensional) system of ordinary differential equations, a further advantage of such an explicit formula is that it may be used to establish qualitative results about the effects of model assumptions. Specifically, we investigate the effect of increasing heterogeneity upon the persistence time of infection in the population.

The remainder of the paper is structured as follows. In Sect. 2, we define precisely our heterogeneous population SIS model, and describe how it may be interpreted as approximating a directed network model. In Sect. 3 we recall some general theory that will be required in the sequel. Our main result, Theorem 1, is derived in Sect. 4, establishing explicit asymptotic formulae for $\ln \tau$ in the large-population limit, provided that heterogeneity is in either infectivity or susceptibility, but not both. Using these explicit formulae, we go on in Sect. 5 to demonstrate that the greater the level of heterogeneity in either infectivity or susceptibility, the more rapidly extinction of infection will occur (on average, to leading order in a large population). In the case that both heterogeneities are present simultaneously, we present an approximate formula for $\lim _{N \rightarrow \infty } ( \ln \tau ) / N$, valid provided $R_0$ is only slightly greater than 1, together with numerical work (Fig. 2) indicating that mean persistence time is again maximised in the homogeneous-population case. In Sect. 6 we demonstrate that if heterogeneity is in susceptibilities, our asymptotic formula for $\ln \tau$, and hence also our conclusion that greater heterogeneity reduces mean persistence time, remain valid for more general infectious period distributions than the classic exponential distribution. We present numerical evidence (Fig. 3) suggesting that this is also true when instead heterogeneity is in infectivities. Finally, in Sect. 7, we discuss the directed network interpretation of our results (Theorem 4) and suggest some directions for further work.

## The SIS infection model in a heterogeneous population or directed network

We first formulate our model in terms of a population divided into a fixed number of groups, and then describe how the same model may be interpreted as modelling an infection spreading on a directed network.

Consider a closed population of *N* individuals divided into *k* groups, with group *i* ($i=1,2,\ldots ,k$) consisting of $N_i$ individuals. Denote by $f_i = N_i / N$ the proportion of the population belonging to group *i*, so that $\sum _i f_i = 1$. When a group *i* individual becomes infected, it remains so for a time distributed as an exponential random variable with mean $1/\gamma$ (assumed for simplicity to be the same for each group). During this infectious period, the group *i* infective makes contacts with each individual in each group $j = 1 , 2 , \ldots , k$ at the points of a Poisson process of rate $\beta \lambda _i \mu _j / N$, where $\beta$ is some overall measure of infectiousness, $\lambda _i$ represents the infectivity of group *i* individuals, and $\mu _j$ represents the susceptibility of group *j* individuals. (The assumption that the group *i* to group *j* infection rate factorises in this way is sometimes referred to as ‘separable mixing’.) Without loss of generality, we scale the $\lambda _i$, $\mu _j$ values so that $\sum _i \lambda _i f_i = \sum _j \mu _j f_j = 1$. These Poisson processes and infectious periods are all mutually independent. If a contacted individual is susceptible, then it becomes infected (and infectious); if the contacted individual is already infected then the contact has no effect. Denoting by $I_j (t)$ the number of infected individuals in group *j* at time *t*, then the process $\left\{ {\varvec{I}}(t) = \left( I_1 (t) , I_2(t) , \ldots , I_k (t) \right) : t \ge 0 \right\}$ is a continuous-time Markov chain with transition rates given in Table 1, and the number of susceptible individuals in group *j* at time $t \ge 0$ is $S_j (t) = N_j - I_j (t)$. We will assume throughout that $\beta , \gamma > 0$, and that $f_i , \lambda _i , \mu _i > 0$ for all *i*. Note that our model is a special case of the model of Clancy and Pearce (2013), although we use slightly different notation here. The basic reproduction number $R_0$ is given by the dominant eigenvalue of the matrix *M* with entries $m_{ij} = \beta \lambda _i \mu _j f_j$, so that

$$\begin{aligned} R_0 = {\beta \over \gamma } \sum _{i=1}^k \lambda _i \mu _i f_i . \end{aligned}$$

**Table 1:** Transition rates for the *k*-group SIS model

Event	State transition	Transition rate
Infection in group *j*	$I_j \rightarrow I_j + 1$	${\beta \over N} \left( \sum _{m=1}^k \lambda _m I_m \right) \mu _j (N_j - I_j)$
Recovery in group *j*	$I_j \rightarrow I_j - 1$	$\gamma I_j$

We now describe how the above model may be interpreted as describing infection spreading through a network. Each of the *N* individuals in the population is assigned an in-degree $d_{\mathrm{in}}$ and out-degree $d_{\mathrm{out}}$ according to some joint probability mass function $p \left( d_{\mathrm{in}},d_{\mathrm{out}} \right)$ on ${\mathbb {Z}}_+^2$. These degrees are assigned independently to distinct individuals, but the in and out degree need not be independent for a single individual. To each individual we attach ‘stubs’, or half-edges, with $d_{\mathrm{in}}$ stubs pointing inwards and $d_{\mathrm{out}}$ stubs pointing outwards. Inward-pointing stubs are then paired with outward-pointing stubs throughout the population, to create links between individuals. In order that this process can produce a valid network, with no left over half-edges, we clearly require that $E \left[ d_{\mathrm{in}} \right] = E \left[ d_{\mathrm{out}} \right]$. We do not concern ourselves with the precise mechanism by which stubs are paired off [see Britton et al. (2007) and Chen and Olvera-Cravioto (2013) for relevant discussion]; rather, we shall simply assume that the resulting network is uncorrelated, so that the so-called ‘annealed’ network approximation is valid for an ensemble of such networks. This is a mean-field approximation for heterogeneous networks, and may be described as follows (see Dorogovtsev et al. 2008 for more comprehensive discussion).

Denote by $\kappa$ the rate at which infection transmits along each link from an infectious individual to a susceptible individual. Suppose for simplicity that there are a finite number *k* of $\left( d_{\mathrm{in}} , d_{\mathrm{out}} \right)$ pairs having non-zero probability, and define a bijective function $c \left( d_{\mathrm{in}} , d_{\mathrm{out}} \right) : {\mathbb {Z}}_+^2 \rightarrow \left\{ 1,2,\ldots ,k \right\}$ that assigns a unique number to each of the possible $\left( d_{\mathrm{in}} , d_{\mathrm{out}} \right)$ pairs. We say an individual is of ‘group *j*’ if they have degrees $\left( d_{\mathrm{in}} , d_{\mathrm{out}} \right) = c^{-1} (j)$, and define $d_{\mathrm{in}} (j) , d_{\mathrm{out}} (j)$ to be the in and out degrees, respectively, of a group *j* individual. For $j = 1,2,\ldots ,k$, denote by $N_j$ the total number of group *j* individuals in the population, and by $I_j (t)$ the number of group *j* individuals who are infectious at time *t*. Then under the annealed network approximation, the total rate at which group *j* individuals become infected is given by

$$\begin{aligned} {\kappa \over N E \left[ d_{\mathrm{in}} \right] } \left( \sum _{m=1}^k d_{\mathrm{out}} (m) I_m \right) d_{\mathrm{in}} (j) \left( N_j - I_j \right) . \end{aligned}$$

When individuals become infected, they remain so for an exponentially distributed time of mean $1/\gamma$ before returning to the susceptible state.

This network model may be approximated by the *k*-group model with transition rates given in Table 1 by taking

$$\begin{aligned} \beta= & {} \kappa E \left[ d_{\mathrm{out}} \right] , \\ f_j= & {} p \left( c^{-1} (j) \right) , \\ \mu _j= & {} d_{\mathrm{in}} (j) / E \left[ d_{\mathrm{in}} \right] , \\ \lambda _j= & {} d_{\mathrm{out}} (j) / E \left[ d_{\mathrm{out}} \right] , \end{aligned}$$

for $$j=1,2,\ldots ,k$$.

The undirected version of the above annealed network approximation (with ${{\varvec{\lambda }}} = {{\varvec{\mu }}}$) has been studied by Hindes and Schwartz (2016), via numerical solution of Hamilton’s equations of motion, Eqs. (15,16) below. We note that real-world social networks are often only partially directed, containing both directed and undirected edges (that is to say, bi-directional edges are over-represented compared to what would be expected by chance), see Spricer and Britton (2015). Each individual then has three-dimensional degree $\left( d_{\mathrm{in}} , d_{\mathrm{out}} , d_{\mathrm{un}} \right)$, where $d_{\mathrm{un}}$ is the number of undirected edges connected to the individual in question. The above annealed network approximation will remain valid if we replace $\left( d_{\mathrm{in}} , d_{\mathrm{out}} \right)$ with $\left( d_{\mathrm{in}} + d_{\mathrm{un}} , d_{\mathrm{out}} + d_{\mathrm{un}} \right)$, provided, as before, that the resulting network is uncorrelated.

In the next section we present some relevant general theory, before going on in Sect. 4 to apply these general methods to the model described above.

## General theory regarding persistence time from endemicity

Consider an infection modelled by a continuous-time Markov process $\left\{ \varvec{X}(t) : t \ge 0 \right\}$ on finite state-space $S \subset {\mathbb {Z}}^k$ with transition rate matrix *Q*. Suppose that *S* is made up of an absorbing set of states *A* (corresponding to absence of disease) and a single transient communicating class *C*. We denote by $Q_C$ the transition rate matrix restricted to *C*. Then the infection will almost surely die out (i.e. the process will leave *C*) within finite time, and (Darroch and Seneta 1967) there exists a unique quasi-stationary distribution ${\varvec{q}}= \left\{ q_{{{\varvec{x}}}} : {{\varvec{x}}} \in C \right\}$ such that, for any initial state within *C*,

$$\begin{aligned} q_{{{\varvec{x}}}} = \lim _{t \rightarrow \infty } \Pr \left( \varvec{X}(t) = {{\varvec{x}}} \left| \varvec{X}(t) \in C \right. \right) \quad \hbox { for } {{\varvec{x}}} \in C . \end{aligned}$$

That is, provided the infection does not die out, it will settle to the endemic distribution ${\varvec{q}}$. The distribution ${\varvec{q}}$ may be found as the unique solution of

3 $$\begin{aligned} {\varvec{q}}Q_C = - (1/\tau ) {\varvec{q}}\hbox { with } \sum _{{{\varvec{x}}} \in C} q_{{{\varvec{x}}}} = 1 , \end{aligned}$$

where $-(1/\tau )$ is the eigenvalue of $Q_C$ with largest real part. The time to extinction from quasi-stationarity is exponentially distributed with mean $\tau$.

Although $\tau$ may be computed exactly from Eq. (3), this can become impractical when the state-space is large, and it is not straightforward from (3) to establish qualitative results. Approximation methods are therefore valuable, and in particular, methods from Hamiltonian statistical mechanics may be used to study the leading order asymptotic (large population) behaviour of $\tau$, as follows.

Suppose that $\varvec{X}(t)$ is a density-dependent process in the sense of chapter 11 of Ethier and Kurtz (2005); that is, the transition rates are of the form

4 $$\begin{aligned} P \left( \varvec{X}( t + \delta t ) = {{\varvec{x}}} + {{\varvec{l}}} \mid \varvec{X}(t) = {{\varvec{x}}} \right) = N W_{{{\varvec{l}}}} \left( {{{\varvec{x}}} \over N} \right) + o ( \delta t ) \quad \hbox {for } {{\varvec{x}}} \in S,\ {{\varvec{l}}} \in L , \end{aligned}$$

for some functions $W_{{{\varvec{l}}}} : {\mathbb {R}}^k \rightarrow {\mathbb {R}}^+$, where *L* is the set of possible jumps from each state ${{\varvec{x}}} \in S$ and *N* is some parameter indicating overall size of the system (in our applications, *N* will be the size of the population). Under mild technical conditions (Ethier and Kurtz 2005, Theorem 11.2.1), the scaled process $\varvec{X}(t) / N$ converges almost surely over finite time intervals, as $N \rightarrow \infty$, to the solution ${{\varvec{y}}} (t)$ of the ordinary differential equation system

5 $$\begin{aligned} {d {{\varvec{y}}} \over dt} = \sum _{{{\varvec{l}}} \in L} {{\varvec{l}}} W_{{{\varvec{l}}}} ( {{\varvec{y}}} ) . \end{aligned}$$

For our application, we suppose that the system (5) possesses two equilibrium points: a stable endemic equilibrium point ${{\varvec{y}}}^*$ with all components strictly positive, and an unstable disease-free equilibrium point at ${{\varvec{y}}} = \mathbf{0}$. We next summarise some key results from the Hamiltonian approach, in a form suited to our application. Detailed justifications and extensions of the method may be found in the review paper (Assaf and Meerson 2017) and references therein.

The Hamiltonian of the system is defined to be

6 $$\begin{aligned} H ( {{\varvec{y}}} , {\varvec{\theta }} ) = \sum _{{{\varvec{l}}} \in L} W_{{{\varvec{l}}}} ( {{\varvec{y}}} ) \left( {\mathrm{e}}^{{\varvec{\theta }}^T {{\varvec{l}}}} - 1 \right) . \end{aligned}$$

This Hamiltonian determines the following two complementary Hamilton–Jacobi partial differential equations:

7 $$\begin{aligned} H \left( {{\varvec{y}}} , {\partial V \over \partial {{\varvec{y}}}} \right) = 0 \hbox { and } H \left( {\partial U \over \partial {\varvec{\theta }}} , {\varvec{\theta }} \right) = 0 . \end{aligned}$$

Each of these Hamilton–Jacobi equations is a way of expressing the eigenvector equation (3) while retaining only leading order terms in the limit $N \rightarrow \infty$ (see “Appendix” for a brief outline of the derivations).

If we can solve either of the Hamilton–Jacobi equations (7), the leading-order asymptotic behaviour of the mean time to extinction $\tau$ is given by

8 $$\begin{aligned} \lim _{N\rightarrow \infty } {\ln \tau \over N} = V ( \mathbf{0} ) - V ( {{\varvec{y}}}^* ) = U ( \mathbf{0} ) - U ( {\varvec{\theta }}^* ) , \end{aligned}$$

where ${{\varvec{y}}}^*$ is the endemic equilibrium point of the deterministic system (5), and ${\varvec{\theta }}^*$ is the (assumed unique) non-zero equilibrium point of the complementary system

9 $$\begin{aligned} {d{\varvec{\theta }} \over dt} = - \left. {\partial H \over \partial {{\varvec{y}}}} \right| _{{{\varvec{y}}} = \mathbf{0}} . \end{aligned}$$

Note that system (5) may be recovered as ${d{{\varvec{y}}} \over dt} = \left. {\partial H \over \partial {\varvec{\theta }}} \right| _{{\varvec{\theta }} = \mathbf{0}}$.

The solutions $U ( {\varvec{\theta }} )$, $V ( {{\varvec{y}}} )$ to equations (7) are related via the Legendre transform; that is,

$$\begin{aligned} U ( {\varvec{\theta }} ) = \sup _{{{\varvec{y}}}} \left\{ {{\varvec{y}}}^T {\varvec{\theta }} - V ( {{\varvec{y}}} ) \right\} , \quad V ( {{\varvec{y}}} ) = \sup _{{\varvec{\theta }}} \left\{ {\varvec{\theta }}^T {{\varvec{y}}} - U ( {\varvec{\theta }} ) \right\} , \end{aligned}$$

see Masoero (2014).

When (as is usually the case) it is not possible to find an analytical solution to either of the Hamilton–Jacobi equations (7), they may be solved numerically using the method of characteristics. That is, we write down the following 2*k*-dimensional system of ordinary differential equations:

10 $$\begin{aligned} \left. \begin{array}{rcl} \displaystyle {d{{\varvec{y}}} \over dt} &{}=&{}\displaystyle {\partial H \over \partial {\varvec{\theta }}} = \sum _{{{\varvec{l}}} \in L} {{\varvec{l}}} W_{{{\varvec{l}}}} ( {{\varvec{y}}} ) {\mathrm{e}}^{{\varvec{\theta }}^T {{\varvec{l}}}} ,\\ \displaystyle {d{\varvec{\theta }} \over dt} &{}=&{} \displaystyle - \; {\partial H \over \partial {{\varvec{y}}}} = - \sum _{{{\varvec{l}}} \in L} {\partial W_{{{\varvec{l}}}} \over \partial {{\varvec{y}}}} \left( {\mathrm{e}}^{{\varvec{\theta }}^T {{\varvec{l}}}} - 1 \right) , \end{array} \right\} \end{aligned}$$

referred to as the ‘equations of motion’ of the system, and apply an appropriate numerical solver to (10). We then have $\lim _{N\rightarrow \infty } ( \ln \tau ) / N = A$, where *A* is the ‘action’ integral,

11 $$\begin{aligned} A = \int _{-\infty }^\infty {\varvec{\theta }}^T {d{{\varvec{y}}} \over dt} \, dt = - \int _{-\infty }^\infty {{\varvec{y}}}^T {d{\varvec{\theta }} \over dt} \, dt , \end{aligned}$$

the integral in each case being evaluated along a trajectory from $( {{\varvec{y}}}^* , \mathbf{0} )$ to $( \mathbf{0} , {\varvec{\theta }}^* )$. Note that $A = V ( \mathbf{0} ) - V ( {{\varvec{y}}}^* ) = U ( \mathbf{0} ) - U ( {\varvec{\theta }}^* )$.

Having set out the general Hamiltonian approach, we will now apply this technique to the infection model described in Sect. 2 above.

## Asymptotic persistence time formulae

Recall the infection model $\left\{ {\varvec{I}}(t) : t \ge 0 \right\}$ described in Sect. 2, with transition rates given in Table 1. In the large population limit, the scaled infection process ${\varvec{I}}(t)/N$ converges almost surely, over finite time intervals, to the deterministic process ${{\varvec{y}}}(t)$ satisfying the system of ordinary differential equations (5); that is,

12 $$\begin{aligned} {dy_i \over dt} = \beta \left( \sum _{j=1}^k \lambda _j y_j \right) \mu _i ( f_i - y_i ) - \gamma y_i \quad \hbox { for } i=1,2,\ldots ,k. \end{aligned}$$

For $R_0 > 1$ there is a unique non-zero equilibrium point ${{\varvec{y}}}^*$ of the system (12), and it is globally asymptotically stable (Lajmanovich and Yorke 1976). This endemic equilibrium point ${{\varvec{y}}}^*$ is given by (Nold 1980)

13 $$\begin{aligned} y_i^* = {\mu _i f_i D ( {{\varvec{\lambda }}},{{\varvec{\mu }}} ) \over 1 + \mu _i D ( {{\varvec{\lambda }}},{{\varvec{\mu }}} ) } \quad \hbox { for } i=1,2,\ldots ,k , \end{aligned}$$

where $D ( {{\varvec{\lambda }}},{{\varvec{\mu }}} )$ is the unique positive solution of

14 $$\begin{aligned} {\beta \over \gamma } \sum _{j=1}^k {\mu _j f_j \lambda _j \over 1 + \mu _j D ( {{\varvec{\lambda }}},{{\varvec{\mu }}} )} = 1 . \end{aligned}$$

The Hamiltonian (6) corresponding to the process ${\varvec{I}}(t)$ is

$$\begin{aligned} H ( {{\varvec{y}}} , {\varvec{\theta }} )= & {} \beta \left( \sum _{j=1}^k \lambda _j y_j \right) \left( \sum _{i=1}^k \mu _i ( f_i - y_i ) \left( {\mathrm{e}}^{\theta _i} - 1 \right) \right) + \gamma \sum _{i=1}^k y_i \left( {\mathrm{e}}^{-\theta _i} - 1 \right) . \end{aligned}$$

The corresponding equations of motion (10) are, for $i=1,2,\ldots ,k$,

15 $$\begin{aligned} {dy_i \over dt}= & {} \beta \left( \sum _{j=1}^k \lambda _j y_j \right) \mu _i ( f_i - y_i ) {\mathrm{e}}^{\theta _i} - \gamma y_i {\mathrm{e}}^{-\theta _i} , \end{aligned}$$

16 $$\begin{aligned} {d\theta _i \over dt}= & {} - \beta \lambda _i \sum _{j=1}^k \mu _j ( f_j - y_j ) \left( {\mathrm{e}}^{\theta _j} - 1 \right) + \beta \left( \sum _{j=1}^k \lambda _j y_j \right) \mu _i \left( {\mathrm{e}}^{\theta _i} - 1 \right) \nonumber \\&- \,\gamma \left( {\mathrm{e}}^{-\theta _i} - 1 \right) . \end{aligned}$$

The non-zero equilibrium point ${\varvec{\theta }}^*$ given by (9) satisfies

17 $$\begin{aligned} \beta \lambda _i \sum _{j=1}^k \mu _j f_j \left( {\mathrm{e}}^{\theta _j^*} - 1 \right) + \gamma \left( {\mathrm{e}}^{-\theta _i^*} - 1 \right) = 0 \quad \hbox { for } i=1,2,\ldots ,k. \end{aligned}$$

Setting $B = (\beta /\gamma ) \sum _j f_j \mu _j \left( 1 - {\mathrm{e}}^{\theta _j^*} \right)$, then (17) implies that

$$\begin{aligned} {\mathrm{e}}^{-\theta _i^*}= & {} 1 + \lambda _i B \quad \hbox { for } i=1,2,\ldots ,k. \end{aligned}$$

Substituting back into Eq. (17), we find that either $B=0$ (corresponding to ${\varvec{\theta }} = \mathbf{0}$) or $B = D ( {{\varvec{\mu }}} , {{\varvec{\lambda }}} )$. The elements of ${\varvec{\theta }}^*$ are thus

$$\begin{aligned} \theta _i^* = - \ln \left( 1 + \lambda _i D ( {{\varvec{\mu }}} , {{\varvec{\lambda }}} ) \right) \quad \hbox { for } i=1,2,\ldots ,k . \end{aligned}$$

So far, we have allowed for heterogeneities in both infectivity and susceptibility simultaneously. If we restrict to only one type of heterogeneity, then it becomes possible to find an explicit formula for the action *A*. Our main result is the following.

### Theorem 1

Consider the heterogeneous SIS infection model defined in Sect. 2, with transition rates given in Table 1, and suppose $R_0 > 1$. Recall that $\tau$ denotes the mean time from quasi-stationarity to disease extinction, and that $D ( {{\varvec{\lambda }}} , {{\varvec{\mu }}} )$ is defined to be the unique positive solution of Eq. (14).

(i) If heterogeneity is in infectivity alone (${{\varvec{\mu }}} = \mathbf{1}$), then
  18 $$\begin{aligned} \lim _{N \rightarrow \infty } {\ln \tau \over N} = \sum _{i=1}^k f_i \ln \left( 1 + \lambda _i D ( \mathbf{1} , {{\varvec{\lambda }}} ) \right) - {\gamma \over \beta } D ( \mathbf{1} , {{\varvec{\lambda }}} ) . \end{aligned}$$
(ii) If heterogeneity is in susceptibility alone (${{\varvec{\lambda }}} = \mathbf{1}$), then
  $$\begin{aligned} \lim _{N \rightarrow \infty } {\ln \tau \over N} = \sum _{i=1}^k f_i \ln \left( 1 + \mu _i D ( \mathbf{1} , {{\varvec{\mu }}} ) \right) - {\gamma \over \beta } D ( \mathbf{1} , {{\varvec{\mu }}} ) . \end{aligned}$$

(Note: under the network interpretation, the assumption ${{\varvec{\mu }}} = \mathbf{1}$ corresponds to every individual having the same in-degree, whereas ${{\varvec{\lambda }}} = \mathbf{1}$ corresponds to every individual having the same out-degree.)

### Proof

(i) Suppose that $\mu _i = 1$ for all *i*, and consider a trajectory $\{ {\varvec{\theta }}(z) : 0 \le z \le D ( \mathbf{1} , {{\varvec{\lambda }}} ) \}$ along which $\theta _i = - \ln \left( 1 + \lambda _i z \right)$ for $i=1,2,\ldots ,k$. Along such a trajectory, the Hamiltonian simplifies to
  $$\begin{aligned} H ( {{\varvec{y}}} , {\varvec{\theta }} )= & {} \beta \left( \sum _{j=1}^k \lambda _j y_j \right) \left( \sum _{i=1}^k ( f_i - y_i ) \left( {1 \over 1 + \lambda _i z} - 1 \right) \right) + \gamma \sum _{i=1}^k y_i \lambda _i z \\= & {} \gamma z \left( \sum _{j=1}^k \lambda _j y_j \right) \left( 1 - {\beta \over \gamma } \sum _{i=1}^k {f_i \lambda _i \over 1 + \lambda _i z} + {\beta \over \gamma } \sum _{i=1}^k {y_i \lambda _i \over 1 + \lambda _i z}\right) . \end{aligned}$$
  Since $z>0$ and $\sum _j \lambda _j y_j >0$ (except at endpoints of the trajectory) the Hamilton–Jacobi equation $H \left( {\partial U \over \partial {\varvec{\theta }}} , {\varvec{\theta }} \right) = 0$ reduces to
  19 $$\begin{aligned} 1 - {\beta \over \gamma } \sum _{i=1}^k {f_i \lambda _i \over 1 + \lambda _i z} + {\beta \over \gamma } \sum _{i=1}^k {\lambda _i \over 1 + \lambda _i z} {\partial U \over \partial \theta _i}= & {} 0 . \end{aligned}$$
  Now along the trajectory under consideration, we have
  $$\begin{aligned} {dU \over dz} = - \sum _{i=1}^k {\lambda _i \over 1 + \lambda _i z} {\partial U \over \partial \theta _i} \end{aligned}$$
  and so Eq. (19) becomes
  $$\begin{aligned} {dU \over dz}= & {} {\gamma \over \beta } - \sum _{i=1}^k {f_i \lambda _i \over 1 + \lambda _i z} \\ \Rightarrow \quad U( {\varvec{\theta }}(z)) - U({\varvec{\theta }}(0))= & {} \left( {\gamma \over \beta } \right) z - \sum _{i=1}^k \int _0^z {f_i \lambda _i \over 1 + \lambda _i x} \, dx \\ \Rightarrow \quad U ( {\varvec{\theta }} ) - U ( \mathbf{0} )= & {} \left( {\gamma \over \beta } \right) z - \sum _{i=1}^k f_i \ln \left( 1 + \lambda _i z \right) \\ \hbox { along } \theta _i= & {} - \ln ( 1 + \lambda _i z ) . \end{aligned}$$
  The action is therefore given by
  $$\begin{aligned} A = U ( \mathbf{0} ) - U ( {\varvec{\theta }}^* ) = \sum _{i=1}^k f_i \ln \left( 1 + \lambda _i D ( \mathbf{1} , {{\varvec{\lambda }}} ) \right) - {\gamma \over \beta } D ( \mathbf{1} , {{\varvec{\lambda }}} ) , \end{aligned}$$
  as required.
(ii) For the SIS model on a finite network, in which each individual *u* makes contact with each other individual *v* at rate $\beta _{uv}$, it is known that, provided infectious periods are exponentially distributed, the decay parameter of the process is unchanged under transposition of the matrix of infection rates $\left\{ \beta _{uv} \right\}$. This follows from the property of ‘network duality’, see Wilkinson and Sharkey (2013), Holley and Liggett (1975) and Harris (1976). In our context, this implies that the mean time to extinction from quasi-stationarity, $\tau$, is identical if we interchange the roles of ${{\varvec{\lambda }}} , {{\varvec{\mu }}}$. Hence part (ii) of the theorem follows immediately from part (i). We can confirm this as follows. With ${{\varvec{\lambda }}} = \mathbf{1}$, the Hamiltonian may be written as
  $$\begin{aligned} H ( {{\varvec{y}}} , {\varvec{\theta }} ) = \gamma \sum _{i=1}^k \left( {\mathrm{e}}^{\theta _i} - 1 \right) \left( {\beta \over \gamma } \left( \sum _{j=1}^k y_j \right) \mu _i ( f_i - y_i ) - y_i {\mathrm{e}}^{-\theta _i} \right) . \end{aligned}$$
  With the convention that $y \ln y = 0$ when $y=0$, take
  20 $$\begin{aligned} V ( {{\varvec{y}}} )= & {} \sum _{i=1}^k y_i \left( 1 + \ln y_i - \ln \left( {\beta \over \gamma } \mu _i \right) \right) - \left( \sum _{i=1}^k y_i \right) \ln \left( \sum _{i=1}^k y_i \right) \nonumber \\&{} + \,\sum _{i=1}^k (f_i - y_i) \ln (f_i - y_i) . \end{aligned}$$
  Then
  $$\begin{aligned} {\partial V \over \partial y_i}= & {} \ln \left( {y_i \over {\beta \over \gamma } \mu _i (f_i - y_i) \left( \sum _j y_j \right) } \right) \quad \hbox { for } i=1,2,\ldots ,k, \end{aligned}$$
  and so
  $$\begin{aligned} H \left( {{\varvec{y}}} , {\partial V \over \partial {{\varvec{y}}}} \right)= & {} 0 . \end{aligned}$$
  That is, $V ( {{\varvec{y}}} )$ satisfies the relevant Hamilton–Jacobi equation. The action is then given by
  $$\begin{aligned} A = V ( \mathbf{0} ) - V ( {{\varvec{y}}}^* ) = \sum _{i=1}^k f_i \ln \left( 1 + \mu _i D ( \mathbf{1} , {{\varvec{\mu }}} ) \right) - {\gamma \over \beta } D ( \mathbf{1} , {{\varvec{\mu }}} ) . \end{aligned}$$
  As expected, we recover the formula for the case of heterogeneous infectivity, but with the roles of ${{\varvec{\lambda }}} , {{\varvec{\mu }}}$ interchanged. Having found the solution $V({{\varvec{y}}})$ for the case ${{\varvec{\lambda }}} = \mathbf{1}$, we can find the corresponding function $U({\varvec{\theta }})$ as the Legendre transform of $V ( {{\varvec{y}}} )$. For $i=1,2,\ldots ,k$, we have
  $$\begin{aligned} {d \over dy_i} \left( {{\varvec{y}}}^T {\varvec{\theta }} - V ( {{\varvec{y}}} ) \right) = \theta _i - \ln \left( {y_i \over {\beta \over \gamma } \mu _i \left( f_i - y_i \right) \left( \sum _j y_j \right) } \right) , \end{aligned}$$
  and so any stationary point satisfies
  21 $$\begin{aligned} y_i = {{\beta \over \gamma } \mu _i f_i \left( \sum _j y_j \right) {\mathrm{e}}^{\theta _i} \over 1 + {\beta \over \gamma } \mu _i \left( \sum _j y_j \right) {\mathrm{e}}^{\theta _i}} . \end{aligned}$$
  For ${\varvec{\theta }} \in {\mathbb {R}}^k$, define the function $Q ( {{\varvec{\mu }}} , {\varvec{\theta }} )$ to be the solution of
  $$\begin{aligned} {\beta \over \gamma } \sum _{j=1}^k {\mu _j f_j \over {\mathrm{e}}^{-\theta _j} + \mu _j Q ( {{\varvec{\mu }}} , {\varvec{\theta }} )} = 1 . \end{aligned}$$
  Setting $R = ( \beta / \gamma ) \sum _j y_j$ and substituting from (21) into the definition of *R*, we find that $R = Q ( {{\varvec{\mu }}} , {\varvec{\theta }} )$, and hence the function ${{\varvec{y}}}^T {\varvec{\theta }} - V ( {{\varvec{y}}} )$ has a stationary point at
  22 $$\begin{aligned} y_i = {\mu _i f_i {\mathrm{e}}^{\theta _i} Q ( {{\varvec{\mu }}} , {\varvec{\theta }} ) \over 1 + \mu _i {\mathrm{e}}^{\theta _i} Q ( {{\varvec{\mu }}} , {\varvec{\theta }} )} . \end{aligned}$$
  Evaluating the function ${{\varvec{y}}}^T {\varvec{\theta }} - V( {{\varvec{y}}} )$ at the point (22), we find
  23 $$\begin{aligned} U ( {\varvec{\theta }} ) = \sum _{i=1}^k f_i \ln \left( 1 + \mu _i {\mathrm{e}}^{\theta _i} Q ( {{\varvec{\mu }}} , {\varvec{\theta }} ) \right) - {\gamma \over \beta } Q ( {{\varvec{\mu }}} , {\varvec{\theta }} ) \end{aligned}$$
  and can easily verify that the function (23) does indeed satisfy $H \left( {\partial U \over \partial {\varvec{\theta }}} , {\varvec{\theta }} \right) = 0$. Now $Q ( {{\varvec{\mu }}} , \mathbf{0} ) = D ( \mathbf{1} , {{\varvec{\mu }}} )$ and $Q ( {{\varvec{\mu }}} , {\varvec{\theta }}^* ) = 0$, so we once again find that
  $$\begin{aligned} A = U ( \mathbf{0} ) - U ( {\varvec{\theta }}^* ) = \sum _{i=1}^k f_i \ln \left( 1 + \mu _i D ( \mathbf{1} , {{\varvec{\mu }}} ) \right) - {\gamma \over \beta } D ( \mathbf{1} , {{\varvec{\mu }}} ) . \end{aligned}$$

$$\square $$

Although we did not actually need to find the functions $U ( {\varvec{\theta }} ) , V ( {{\varvec{y}}} )$ in order to prove Theorem 1(ii), we include them because knowledge of these functions can be of assistance in generalising and extending our results. We will demonstrate this in Theorem 3 below.

**Fig. 1:**
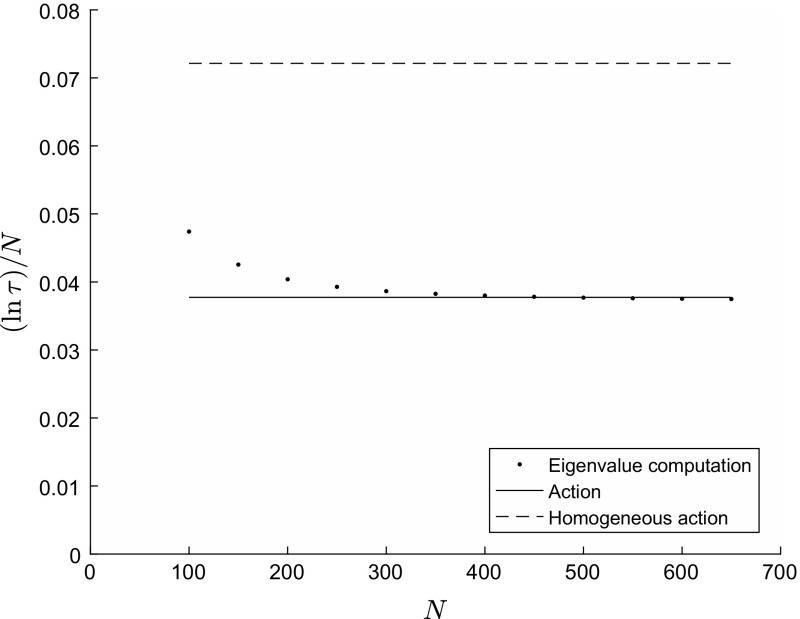
Values of $( \ln \tau ) / N$ and asymptotic formulae plotted against population size *N*. Fixed parameter values $k=2$, $\varvec{f} =(0.5,0.5)$, ${{\varvec{\lambda }}} = {2 \over 51} (50,1)$, ${{\varvec{\mu }}} = (1,1)$, $R_0 = 1.5$, $\gamma = 1$. For these parameter values, $D ( {{\varvec{\lambda }}} , {{\varvec{\mu }}} ) = 0.5$, ${{\varvec{y}}}^* = (1/6 , 1/6)$, $D ( {{\varvec{\mu }}} , {{\varvec{\lambda }}} ) = 0.2625$, ${\varvec{\theta }}^*=(-0.4152 -0.0102)$, with action $A \approx 0.0377$ and corresponding action for the homogeneous case $A_0 \approx 0.0721$. The dots, labelled ‘eigenvalue computation’, are the true values of $( \ln \tau ) / N$ computed from Eq. (3); the action *A* is computed from Eq. (18); the homogeneous action is computed as $A_0 = (1/R_0) - 1 + \ln R_0$

Figure 1 illustrates Theorem 1 in the case of $k=2$ groups with heterogeneity in infectivity (the graph for the corresponding case with heterogeneity in susceptibility is identical, by network duality). The exact value of $( \ln \tau ) / N$ is computed from Eq. (3) for total population sizes $N=100, 150, \ldots , 650$. The action *A* is computed from Eq. (18). For comparison, we also show the action $A_0 = (1/R_0) - 1 + \ln R_0$ computed for the homogeneous population SIS model with the same value for $R_0$. We see that formula (18) gives a good approximation to $( \ln \tau ) / N$ for population sizes from around $N=300$ upwards. We can also see that if we were to ignore heterogeneity and use the homogeneous population result, we would drastically over-estimate the persistence time of infection. We demonstrate this point in Theorem 2 below, as well as comparing heterogeneous populations of greater or lesser degrees of heterogeneity.

## The effect of increasing heterogeneity

Using the formulae of the previous section, we are now in a position to investigate the effect of increasing heterogeneity upon the persistence time of infection. First, in order to compare different levels of heterogeneity, we recall the definition of majorization (Marshall et al. 2011). For any ${{\varvec{x}}} \in ({\mathbb {R}}^+)^k$, denote by $x_{[1]} \ge x_{[2]} \ge \cdots \ge x_{([k]}$ the ordered components of ${{\varvec{x}}}$. Then for ${{\varvec{x}}}^{(1)} , {{\varvec{x}}}^{(2)} \in ({\mathbb {R}}^+)^k$, we say ${{\varvec{x}}}^{(1)}$ is majorized by ${{\varvec{x}}}^{(2)}$, denoted ${{\varvec{x}}}^{(1)} \prec {{\varvec{x}}}^{(2)}$, if $\sum _{i=1}^k x^{(1)}_i = \sum _{i=1}^k x^{(2)}_i$ and $\sum _{i=1}^j x^{(1)}_{[i]} \le \sum _{i=1}^j x^{(2)}_{[i]}$ for $j=1,2,\ldots ,k-1$. An equivalent definition is that $\sum _{i=1}^k \phi \left( x^{(1)}_i \right) \le \sum _{i=1}^k \phi \left( x^{(2)}_i \right)$ for all convex functions $\phi (\cdot )$. Intuitively, ${{\varvec{x}}}^{(2)}$ is ‘more heterogeneous’ than ${{\varvec{x}}}^{(1)}$. More generally, given a probability vector (with components summing to 1) ${\varvec{p}}\in ( {\mathbb {R}}^+ )^k$, then ${{\varvec{x}}}^{(1)}$ is ${\varvec{p}}$-majorized by ${{\varvec{x}}}^{(2)}$, written ${{\varvec{x}}}^{(1)} \prec _{{\varvec{p}}} {{\varvec{x}}}^{(2)}$, if there exists a permutation $\sigma$ such that $x^{(1)}_{\sigma (1)} \ge x^{(1)}_{\sigma (2)} \ge \cdots \ge x^{(1)}_{\sigma (k)}$ and $x^{(2)}_{\sigma (1)} \ge x^{(2)}_{\sigma (2)} \ge \cdots \ge x^{(2)}_{\sigma (k)}$ with $\sum _{i=1}^k p_i x^{(1)}_i = \sum _{i=1}^k p_i x^{(2)}_i$ and $\sum _{i=1}^j p_{\sigma (i)} x^{(1)}_{\sigma (i)} \le \sum _{i=1}^j p_{\sigma (i)} x^{(2)}_{\sigma (i)}$ for $j=1,2,\ldots ,k-1$.

### Theorem 2

Consider two populations, with $\beta ^{(1)} = \beta ^{(2)} = \beta$, $\gamma ^{(1)} = \gamma ^{(2)} = \gamma$, and each having the same group structure ${\varvec{f}}^{(1)} = {\varvec{f}}^{(2)} = {\varvec{f}}$, where we use superscripts (1), (2) to denote the population under consideration. Recall that $\tau$ denotes the mean time from quasi-stationarity to disease extinction.

(i) With heterogeneity in infectivity alone,
  $$\begin{aligned} {{\varvec{\lambda }}}^{(1)} \prec _{{\varvec{f}}} {{\varvec{\lambda }}}^{(2)} \Rightarrow \lim _{N \rightarrow \infty } {\ln \tau ^{(1)} \over N} \ge \lim _{N \rightarrow \infty } {\ln \tau ^{(2)} \over N} . \end{aligned}$$
(ii) With heterogeneity in susceptibility alone,
  $$\begin{aligned} {{\varvec{\mu }}}^{(1)} \prec _{{\varvec{f}}} {{\varvec{\mu }}}^{(2)} \Rightarrow \lim _{N \rightarrow \infty } {\ln \tau ^{(1)} \over N} \ge \lim _{N \rightarrow \infty } {\ln \tau ^{(2)} \over N} . \end{aligned}$$

In particular, provided heterogeneity is in either infectivity or susceptibility but not both, then $\lim _N \rightarrow \infty \left( \ln \tau \right) / N$ is maximised in the homogeneous case.

### Proof

Consider the case of heterogeneity in infectivity, and suppose that ${{\varvec{\lambda }}}^{(1)} \prec _{{\varvec{f}}} {{\varvec{\lambda }}}^{(2)}$. The function $h(x) = x / \left( 1 + x D \left( \mathbf{1} , {{\varvec{\lambda }}}^{(1)} \right) \right)$ is concave for $x>0$, and so applying proposition 14.A.3 of Marshall et al. (2011),

$$\begin{aligned} \sum _{i=1}^k {f_i \lambda _i^{(2)} \over 1 + \lambda _i^{(2)} D ( \mathbf{1} , {{\varvec{\lambda }}}^{(1)} )} \le \sum _{i=1}^k {f_i \lambda _i^{(1)} \over 1 + \lambda _i^{(1)} D ( \mathbf{1} , {{\varvec{\lambda }}}^{(1)} )} = {\gamma \over \beta } , \end{aligned}$$

the final equality coming from the definition (14) of $D ( {{\varvec{\mu }}} , {{\varvec{\lambda }}} )$ for population 1. The expression $\sum _i f_i \lambda _i^{(2)} / \left( 1 + \lambda _i^{(2)} z \right)$ is a decreasing function of *z*, and so from Eq. (14) for population 2 it follows that $D \left( \mathbf{1} , {{\varvec{\lambda }}}^{(1)} \right) \ge D \left( \mathbf{1} , {{\varvec{\lambda }}}^{(2)} \right)$.

Now define the function $\psi (z) = \sum _i f_i \ln \left( 1 + \lambda _i^{(1)} z \right) - (\gamma z / \beta )$. Then

$$\begin{aligned} {d\psi \over dz} = \sum _i {f_i \lambda _i^{(1)} \over 1 + \lambda _i^{(1)} z} - {\gamma \over \beta } , \end{aligned}$$

so that $\psi ^\prime (z) > 0$ for $0< z < D \left( \mathbf{1} , {{\varvec{\lambda }}}^{(1)} \right)$, and hence $\psi \left( D \left( \mathbf{1} , {{\varvec{\lambda }}}^{(2)} \right) \right) \le \psi \left( D \left( \mathbf{1} , {{\varvec{\lambda }}}^{(1)} \right) \right) = A^{(1)}$. That is,

24 $$\begin{aligned} \sum _{i=1}^k f_i \ln \left( 1 + \lambda _i^{(1)} D \left( \mathbf{1} , {{\varvec{\lambda }}}^{(2)} \right) \right) - {\gamma D \left( \mathbf{1} , {{\varvec{\lambda }}}^{(2)} \right) \over \beta } \le A^{(1)} . \end{aligned}$$

The function $g(x) = \ln \left( 1 + D \left( \mathbf{1} , {{\varvec{\lambda }}}^{(2)} \right) x \right)$ is concave for $x>0$, and so again applying proposition 14.A.3 of Marshall et al. (2011),

$$\begin{aligned} \sum _{i=1}^k f_i \ln \left( 1 + \lambda _i^{(2)} D \left( \mathbf{1} , {{\varvec{\lambda }}}^{(2)} \right) \right) \le \sum _{i=1}^k f_i \ln \left( 1 + \lambda _i^{(1)} D \left( \mathbf{1} , {{\varvec{\lambda }}}^{(2)} \right) \right) . \end{aligned}$$

Combining with (24) yields

$$\begin{aligned} A^{(2)} \le A^{(1)} , \end{aligned}$$

and the result follows.

Part (ii) of the theorem follows immediately by interchanging the roles of ${{\varvec{\lambda }}}, {{\varvec{\mu }}}$. $\square$

**Fig. 2:**
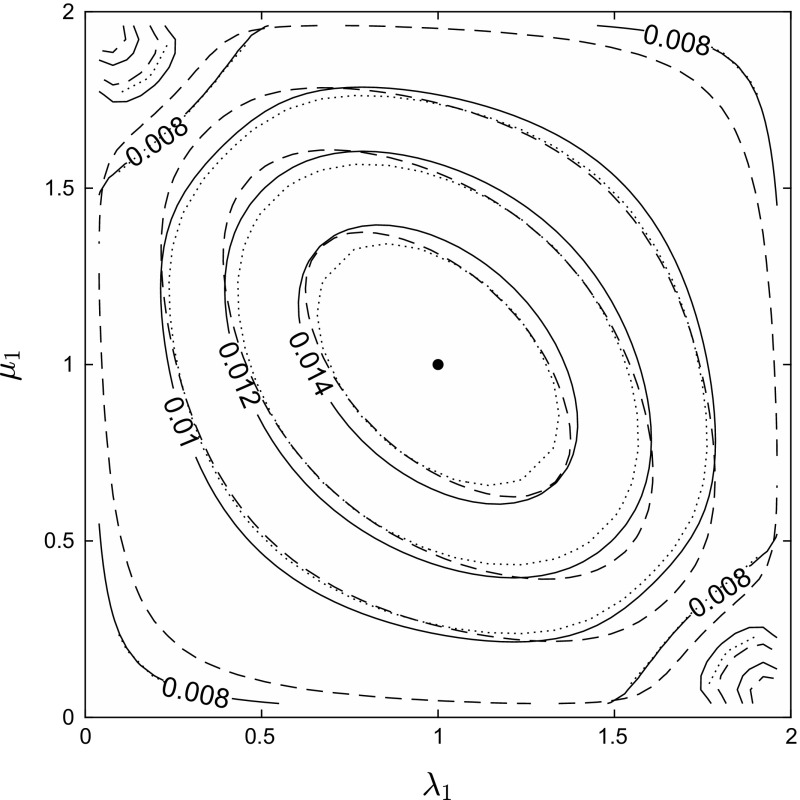
Contour plot showing the action *A* (solid contours) as a function of $\lambda _1 , \mu _1$. Fixed parameter values $k=2$, ${\varvec{f}}=(0.5,0.5)$, $R_0 = 1.2$. The action is maximised at $(\lambda _1 , \mu _1 ) = (1,1)$, with value $A_0 = (1/R_0) - 1 + \ln R_0 \approx 0.0157$. Dashed contours show the approximation $\tilde{A}$ computed from formula (25). Dotted contours show a finite-population approximation—see main text for details

Figure 2 illustrates Theorem 2, as well as showing the effect of allowing heterogeneity in both infectivity and susceptibility simultaneously, for the case of $k=2$ equal-sized groups $(f_1=f_2=0.5)$. The constraints on the elements of ${{\varvec{\lambda }}} , {{\varvec{\mu }}}$ in this case reduce to $\lambda _1 + \lambda _2 = \mu _1 + \mu _2 = 2$, and so we plot the action as a function of $( \lambda _1 , \mu _1 ) \in (0,2)^2$. We choose to keep $R_0$ fixed, with the value of $\beta$ being varied in order to achieve this. With both heterogeneities present, we have no explicit formula for the action *A*, and instead compute it by first solving the equations of motion (15,16) numerically using the Matlab bvp4c command, and then integrating the numerical solution along the trajectory, Eq. (11). The solid contours in Fig. 2 show the action values *A* computed in this way. Note that the transformation $(\lambda _1 , \mu _1 ) \rightarrow ( 2-\lambda _1 , 2-\mu _1 )$ here amounts to simply re-labelling the groups, so that Fig. 2 is invariant under a rotation of half a turn around the point (1, 1); also, we know from network duality that the action is unchanged under the transformation $(\lambda _1 , \mu _1) \rightarrow (\mu _1 , \lambda _1)$, so that Fig. 2 is invariant under reflection in the line $\lambda _1 = \mu _1$.

Although we do not have an explicit formula for *A* when both types of heterogeneity are present, we can obtain an approximate formula valid for $R_0$ close to (and above) 1, as follows. For $R_0$ only slightly larger than 1, the trajectory from $\left( {{\varvec{y}}}^* , \mathbf{0} \right)$ to $\left( \mathbf{0} , {\varvec{\theta }}^* \right)$ satisfying equations (15,16) may be approximated by a straight line. The integral (11) can easily be evaluated along the straight line connecting $\left( {{\varvec{y}}}^* , \mathbf{0} \right)$ to $\left( \mathbf{0} , {\varvec{\theta }}^* \right)$, and we thus find that $A \approx \tilde{A}$, where

25 $$\begin{aligned} \tilde{A} = {D ( {{\varvec{\lambda }}} , {{\varvec{\mu }}} ) \over 2} \sum _{i=1}^k {\mu _i f_i \ln \left( 1 + \lambda _i D ( {{\varvec{\mu }}} , {{\varvec{\lambda }}} ) \right) \over 1 + \mu _i D ( {{\varvec{\lambda }}} , {{\varvec{\mu }}} )} . \end{aligned}$$

Values of $\tilde{A}$ are shown as dashed contours in Fig. 2, and we see that for these parameter values $\tilde{A}$ does indeed provide a reasonable approximation to the true value *A*. Note that although the action *A* is known (from network duality) to be invariant under the interchange of ${{\varvec{\lambda }}} , {{\varvec{\mu }}}$, the approximating formula (25) does not possess this symmetry. Nevertheless, we see that in Fig. 2 the contours corresponding to $\tilde{A}$ do appear symmetrical under reflection in the line $\lambda _1 = \mu _1$, so that the expected symmetry does at least hold approximately here.

For comparison, the dotted contours in Fig. 2 were computed by solving the eigenvalue equation (3) numerically for $N=400$ and $N=500$, and assuming (without proof) that asymptotic formula (1) is valid for our model. Denoting by $\tau _N$ the mean time from quasi-stationarity to disease extinction in a population of size *N*, formula (1) implies that the action *A* may be approximated by

26 $$\begin{aligned} \left( \ln \left( \tau _{500} \sqrt{500} \right) - \ln \left( \tau _{400} \sqrt{400} \right) \right) \Big / 100 , \end{aligned}$$

and the dotted contours show computed values of formula (26). The fact that the dotted contours closely follow the solid contours provides some confirmation both that the action *A* gives a good approximation to $(\ln \tau ) / N$ for population sizes above $N=400$, and that formula (1) does indeed apply to our model.

We see from Fig. 2 that the action decreases as we move away from the point $( \lambda _1 , \mu _1 ) = (1,1)$, not only along the lines $\lambda _1 = 1$ and $\mu _1 = 1$, as ensured by Theorem 2, but in any direction. That is, heterogeneity in infectivity, or susceptibility, or any combination of the two, reduces the value of $\lim _{N \rightarrow \infty } ( \ln \tau ) / N$ compared to the homogeneous case. We discuss this further in Sect. 7 below.

## Generalising the infectious period distribution

So far, we have made the conventional assumption that individuals’ infectious periods are exponentially distributed. This is purely a mathematical convenience, not motivated by biological realism. Realism can be greatly improved by allowing infectious periods to follow an Erlang distribution, using the ‘method of stages’. That is, when an individual becomes infected, it passes through *s* infectious stages, remaining in each stage for an exponentially distributed time of mean $(s\gamma )^{-1}$, before returning to susceptibility. As before, we denote by $N_j$ the (constant) number of individuals in group *j*, and by $N = N_1 + N_2 +\cdots + N_k$ the total population size. Denoting by $I_{jv} (t)$ the number of group *j* individuals in infectious stage *v* at time *t*, then $\left\{ I_{jv} (t) : j=1,2,\ldots ,k, \ v=1,2,\ldots ,s,\ t \ge 0 \right\}$ is a continuous-time Markov chain with transition rates given in Table 2. The number of susceptible individuals in group *j* is $S_j (t) = N_j - \sum _{v=1}^s I_{jv} (t)$.

**Table 2:** Transition rates for the *k*-group, *s*-stage SIS model

Event	State transition	Transition rate
Infection in group *j*	$I_{j1} \rightarrow I_{j1} + 1$	${\beta \over N} \left( \sum _{m=1}^k \lambda _m \sum _{v=1}^s I_{mv} \right) \mu _j \left( N_j - \sum _{v=1}^s I_{jv} \right)$
Transition to next infectious stage	$\left( I_{j,v-1} , I_{jv} \right) \rightarrow ( I_{j,v-1} - 1 , I_{jv} + 1)$	$s \gamma I_{j,v-1}$ for $v=2,3,\ldots ,s$
Recovery in group *j*	$I_{js} \rightarrow I_{js} - 1$	$s \gamma I_{js}$

Writing ${{\varvec{y}}} = \left\{ y_{iv} : i=1,2,\ldots ,k,\ v=1,2,\ldots ,s \right\}$ and ${\varvec{\theta }} = \{ \theta _{iv} : i=1,2,\ldots ,k,\ v=1,2,\ldots ,s \}$, the corresponding Hamiltonian is

$$\begin{aligned} H ( {{\varvec{y}}} , {\varvec{\theta }} )= & {} \beta \left( \sum _{j=1}^k \lambda _j \sum _{v=1}^s y_{jv} \right) \left( \sum _{i=1}^k \mu _i \left( f_i - \sum _{v=1}^s y_{iv} \right) \right) \left( {\mathrm{e}}^{\theta _{i1}} - 1 \right) \\&+\, s \gamma \sum _{i=1}^k \sum _{v=1}^{s-1} y_{iv} \left( {\mathrm{e}}^{-\theta _{iv}+\theta _{i,v+1}} - 1 \right) + s \gamma \sum _{i=1}^k y_{is} \left( {\mathrm{e}}^{-\theta _{is}} - 1 \right) . \end{aligned}$$

It is immediate from equation (17) of Clancy (2015) that the deterministic endemic equilibrium point is given by

$$\begin{aligned} y_{iv}^* = {y_i^* \over s} \quad \hbox { for } i=1,2,\ldots ,k,\ v=1,2,\ldots ,s, \end{aligned}$$

where $y_i^*$ is the solution (13) for the model with exponentially distributed infectious periods ($s=1$).

It is straightforward to show that the elements of ${\varvec{\theta }}^*$ are given by

$$\begin{aligned} \theta _{iv}^* = - (s+1-v) \ln \left( 1 + \lambda _i D_s ( {{\varvec{\mu }}} , {{\varvec{\lambda }}} ) \right) \end{aligned}$$

where $D_s ( {{\varvec{\mu }}} , {{\varvec{\lambda }}} )$ is the solution of

27 $$\begin{aligned} {\beta \over s \gamma } \sum _{j=1}^k \mu _j f_j \lambda _j \sum _{v=1}^s \left( {1 \over 1 + \lambda _j D_s ( {{\varvec{\mu }}} , {{\varvec{\lambda }}} )} \right) ^v = 1 . \end{aligned}$$

For the SIS model with Erlang-distributed infectious periods in a homogeneous population ($k=1$), the solution $U ( {\varvec{\theta }} )$ to the relevant Hamilton–Jacobi equation was found in Clancy and Tjia (2018) to be

$$\begin{aligned} U ( {\varvec{\theta }} ) = \ln \left( \sum _{v=1}^s {\mathrm{e}}^{\theta _v} \right) + {\gamma \over \beta } \left( {s \over \sum _{v=1}^s {\mathrm{e}}^{\theta _v}} \right) . \end{aligned}$$

Taking the Legendre transform, we find that $V ( {{\varvec{y}}} )$ for this homogeneous-population model is given by

28 $$\begin{aligned} V ( {{\varvec{y}}} )= & {} \sup _{{\varvec{\theta }}} \left\{ {\varvec{\theta }}^T {{\varvec{y}}} - U ( {\varvec{\theta }} ) \right\} \nonumber \\= & {} \sum _{v=1}^s y_v \left( 1 + \ln y_v - \ln \left( {\beta \over s \gamma } \right) \right) - \left( \sum _{v=1}^s y_v \right) \ln \left( \sum _{v=1}^s y_v \right) \nonumber \\&+\, \left( 1 - \sum _{v=1}^s y_v \right) \ln \left( 1 - \sum _{v=1}^s y_v \right) . \end{aligned}$$

Comparing solution (28) for the SIS model with Erlang-distributed infectious periods in a homogeneous population and solution (20) for the SIS model with exponentially distributed infectious periods and heterogeneous susceptibilities, one may now guess the form of the solution $V ( {{\varvec{y}}} )$ for the SIS model with Erlang-distributed infectious periods and heterogeneous susceptibilities, and verify that the relevant Hamilton–Jacobi equation is indeed satisfied. The solution is thus found to be

29 $$\begin{aligned} V ( {{\varvec{y}}} )= & {} \sum _{i=1}^k \sum _{v=1}^s y_{iv} \left( 1 + \ln y_{iv} - \ln \left( {\beta \over s\gamma } \mu _i \right) \right) - \left( \sum _{i=1}^k \sum _{v=1}^s y_{iv} \right) \ln \left( \sum _{i=1}^k \sum _{v=1}^s y_{iv} \right) \nonumber \\&{} + \,\sum _{i=1}^k \left( f_i - \sum _{v=1}^s y_{iv} \right) \ln \left( f_i - \sum _{v=1}^s y_{iv} \right) . \end{aligned}$$

Taking the Legendre transform, we find

$$\begin{aligned} U ( {\varvec{\theta }} ) = \sum _{i=1}^k f_i \ln \left( 1 + \mu _i \left( \sum _{v=1}^s {\mathrm{e}}^{\theta _{iv}} \right) Q_s ( {{\varvec{\mu }}} , {\varvec{\theta }} ) \right) - {\gamma s \over \beta } Q_s ( {{\varvec{\mu }}} , {\varvec{\theta }} ) \end{aligned}$$

where $Q_s ( {{\varvec{\mu }}}, {\varvec{\theta }} )$ is the solution of

$$\begin{aligned} {\beta \over \gamma s} \sum _{j=1}^k {\mu _j f_j \left( \sum _{v=1}^s {\mathrm{e}}^{\theta _{jv}} \right) \over 1 + \mu _j \left( \sum _{v=1}^s {\mathrm{e}}^{\theta _{jv}} \right) Q_s ( {{\varvec{\mu }}} , {\varvec{\theta }} )} = 1 . \end{aligned}$$

The action *A* in this case is thus

$$\begin{aligned} A= & {} V ( \mathbf{0} ) - V ( {{\varvec{y}}}^* ) \\= & {} U ( \mathbf{0} ) - U ( {\varvec{\theta }}^* ) = \sum _i f_i \ln \left( 1 + \mu _i D ( \mathbf{1} , {{\varvec{\mu }}} ) \right) - {\gamma \over \beta } D ( \mathbf{1} , {{\varvec{\mu }}} ) , \end{aligned}$$

as before, and the following result is immediate.

### Theorem 3

Theorem 1(ii) remains valid if infectious periods are allowed to follow an Erlang, rather than exponential, distribution. Consequently, Theorem 2(ii) likewise remains valid with Erlang-distributed infectious periods.

**Fig. 3:**
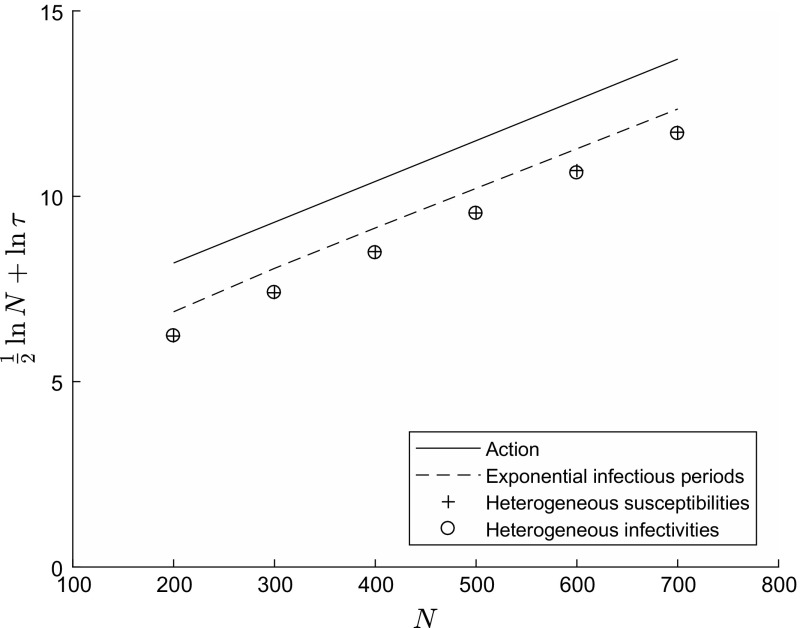
The effect of the infectious period distribution upon the mean persistence time of infection $\tau$. Fixed parameter values $k=2$, ${\varvec{f}} = (0.5,0.5)$, $R_0 = 1.2$, $\gamma = 1$. Solid line (‘Action’) has gradient $A\approx 0.0110$ given by Eq. (18) with ${{\varvec{\lambda }}} = (5/3,1/3)$, ${{\varvec{\mu }}} = (1,1)$, intercept chosen arbitrarily; dashed line (‘Exponential infectious periods’) computed from Eq. (3) with ${{\varvec{\lambda }}} = (5/3,1/3)$, ${{\varvec{\mu }}} = (1,1)$; crosses (‘Heterogeneous susceptibilities’) computed via simulation with ${{\varvec{\lambda }}} = ( 1,1 )$, ${{\varvec{\mu }}} = (5/3,1/3)$ and constant infectious periods; circles (‘Heterogeneous infectivities’) computed via simulation with ${{\varvec{\lambda }}} = (5/3,1/3)$, ${{\varvec{\mu }}} = (1,1)$ and constant infectious periods

Figure 3 illustrates the effect of the infectious period distribution in the case of $k=2$ equal-sized groups ($f_1 = f_2 = 0.5$). In constructing this figure, we have assumed (without proof) that asymptotic formula (1) is valid for our model. Consequently, we plot the function ${1 \over 2} \ln N + \ln \tau$, which according to formula (1) should, as *N* increases, approach a straight line of gradient *A* and intercept $\ln C$. The dashed line, corresponding to exponentially distributed infectious periods, was computed using the eigenvalue characterisation (3). By network duality, this dashed line may be interpreted as corresponding to either heterogeneous infectivity (${{\varvec{\lambda }}} = (5/3,1/3)$, ${{\varvec{\mu }}} = (1,1)$) or heterogeneous susceptibility (${{\varvec{\lambda }}} = (1,1)$, ${{\varvec{\mu }}} = (5/3,1/3)$). We used Monte Carlo simulation to estimate the mean persistence time $\tau$ with constant (non-random) infectious periods for the cases of heterogeneous infectivity and heterogeneous susceptibility separately. This infectious period distribution corresponds to an Erlang distribution with *s* stages in the limit as $s \rightarrow \infty$. An issue that arises is that the time until extinction of infection, starting from quasi-stationarity, is exponentially distributed with mean increasing exponentially in population size, so that to simulate the process to extinction can be very time-consuming. To get around this, we fixed times $t_0$ (the burn-in period) and $t_{max}$ such that (i) by time $t_0$ the state of the process is approximately quasi-stationary (having started at time zero close to the re-scaled deterministic equilibrium point $N {{\varvec{y}}}^*$, and conditioning upon survival to time $t_0$); and (ii) by time $t_{max}$ a substantial proportion of all simulations have reached extinction. We then estimated the mean time to extinction $\tau$ using the maximum likelihood estimator. That is, denoting by $T_1 , T_2 , \ldots , T_r$ the extinction times of those simulations that went extinct within the time window $\left( t_0 , t_{max} \right)$, and by *m* the number of simulations that had not gone extinct by time $t_{max}$, our estimate is

$$\begin{aligned} \hat{\tau } = {m \left( t_{max} - t_0 \right) + \sum _{i=1}^r ( T_i - t_0 ) \over r} . \end{aligned}$$

We have included in Fig. 3 a solid line with gradient equal to the action *A* computed from formula (18). Note that the intercept of this line was chosen arbitrarily, since we have no way to evaluate the constant *C* in formula (1) for our model.

We see from Fig. 3 that the dashed line corresponding to exponentially distributed infectious periods does indeed appear to be a straight line of gradient *A*, providing some confirmation both that the action *A* gives a reasonable approximation to $( \ln \tau ) / N$ for population sizes above $N=200$ and that formula (1) is valid for our model. With constant infectious periods, we see that heterogeneous infectivity and heterogeneous susceptibility result in almost identical estimates of $\tau$, and that these estimates lie close to a straight line of gradient *A*. It appears that, as with exponentially distributed infectious periods, the value of $\tau$ is unchanged if we interchange ${{\varvec{\lambda }}} , {{\varvec{\mu }}}$. We therefore conjecture that, similarly to Theorem 3 above, Theorem 1(i) and Theorem 2(i) remain valid with Erlang-distributed infectious periods. The model with constant infectious periods has reduced mean persistence time $\tau$ compared to the model with exponentially distributed infectious periods, but the difference is in the pre-factor constant *C* and not the leading-order constant *A*, in line with the results of Ball et al. (2016) for the homogeneous population case.

## Discussion and possible extensions

The main result of this paper, Theorem 1, provides a simple explicit formula for $\lim _{N \rightarrow \infty } ( \ln \tau ) / N$, where $\tau$ is the expected time from endemicity to extinction for an SIS infection model with heterogeneity in either infectivity or susceptibility of individuals, in a population of size *N*. The only infection model for which such a formula has previously been available is the SIS model in a homogeneous population, either with exponentially distributed infectious periods (Andersson and Djehiche 1998) or with arbitrary infectious period distribution (Ball et al. 2016). Theorem 1 thus represents a significant advance, but many open questions remain.

Firstly, for the SIS model in a homogeneous population, both of Andersson and Djehiche (1998) and Ball et al. (2016) established asymptotic approximations for $\tau$ of the form (1), with explicit formulae for the pre-factor constant *C*. Our result is less precise than this; we have not shown that an asymptotic formula of the form (1) is valid for our model (although we conjecture, and have presented some numerical evidence, that this is the case), nor have we attempted to evaluate the pre-factor constant *C*. The asymptotic form (1) has been shown by Assaf and Meerson (2010) to be valid (and formulae given for the constant *C*) for general 1-dimensional processes of bounded jump size. The technique of Assaf and Meerson (2010) is an extension of the approach employed here, retaining terms beyond the leading order in *N*. The analysis is considerably more intricate than the leading-order treatment we have restricted ourselves to, and the extension of the approach of Assaf and Meerson (2010) to multi-dimensional processes such as the infection model considered here is the subject of ongoing work.

Secondly, our model as described in Sect. 2 incorporates heterogeneity in both infectivity and susceptibility simultaneously, but we have only been able to provide an explicit asymptotic formula for the cases in which only one of these two heterogeneities is present. When both heterogeneities are present, provided $R_0$ is only slightly above one then $\tilde{A}$ given by formula (25) can be used to approximate $A = \lim _{N \rightarrow \infty } ( \ln \tau ) / N$, and in Fig. 2 we saw that $\tilde{A}$ can indeed provide a reasonable approximation to *A*. However, even a small error in *A* can correspond to a very large error in our estimate of $\tau$, so that an approximate formula such as (25) is of considerably less utility than an exact formula such as (18). In particular, the fact that we have not been able to find an exact formula such as (18) valid when both types of heterogeneity are present severely restricts the class of networks to which our results may be applied under the annealed network approximation—we require either that every individual has the same in-degree, or that every individual has the same out-degree. Nevertheless, for the class of directed networks to which they apply our results represent an interesting step forward, and since network models are of great ongoing interest in infection modelling, we now present our results in a form suited to the network interpretation.

As a preliminary to the statement of our results, we require the concept of convex ordering of random variables, defined as follows (Shaked and Shanthikumar 2007, section 3.A.1). Given two random variables $X^{(1)},X^{(2)}$, then $X^{(2)}$ is greater than $X^{(1)}$ in the sense of convex ordering, denoted $X^{(1)} \le _{cv} X^{(2)}$, if $E \left[ \phi \left( X^{(1)} \right) \right] \le E \left[ \phi \left( X^{(2)} \right) \right]$ for all convex functions $\phi (\cdot )$. If $X^{(1)} , X^{(2)}$ take values in $\left\{ 1,2,\ldots , d_{\max } \right\}$, then an equivalent definition is that

$$\begin{aligned} \sum _{i=1}^j P \left( X^{(1)} \le i \right) \le \sum _{i=1}^j P \left( X^{(2)} \le i \right) \quad \hbox { for } j=1,2,\ldots ,d_{\max } . \end{aligned}$$

Note that $X^{(1)} \le _{cv} X^{(2)}$ implies $E \left[ X^{(1)} \right] = E \left[ X^{(2)} \right]$; intuitively, $X^{(2)}$ is ‘more variable’ than $X^{(1)}$.

In comparing two populations, we need to define our ‘groups’ slightly differently than in Sect. 2. Specifically, partition the population into groups in such a way that two individuals belong to the same group if they share the same values of both $\left( d_{\mathrm{in}}^{(1)} , d_{\mathrm{out}}^{(1)} \right)$ and $\left( d_{\mathrm{in}}^{(2)} , d_{\mathrm{out}}^{(2)} \right)$; the condition ${\varvec{f}}^{(1)} = {\varvec{f}}^{(2)} = {\varvec{f}}$ required by Theorem 2 is thus satisfied. (As before, superscripts (1), (2) denote the population under consideration.)

### Theorem 4

Consider an SIS infection in a population of *N* individuals connected by an uncorrelated directed network. Each individual has in-degree and out-degree distributed as $\left( d_{\mathrm{in}}, d_{\mathrm{out}} \right)$, the degrees of distinct individuals being mutually independent, with $E \left[ d_{\mathrm{in}} \right] = E \left[ d_{\mathrm{out}} \right] = \mu$ and $d_{\mathrm{in}} , d_{\mathrm{out}} \le d_{\max }$ for some $d_{\max } \in {\mathbb {N}}$. Infection transmits along each link from an infectious to a susceptible individual at rate $\kappa$, and when an individual becomes infected it remains so for a time of mean $1/\gamma$ before returning to the susceptible state. Recall that $\tau$ denotes the expected time from quasi-stationarity to extinction of infection.

- Suppose that $P \left( d_{\mathrm{in}} = \mu \right) = 1$, so every individual has the same in-degree, and that infectious periods are exponentially distributed. Then
  (i) $$\displaystyle \lim \nolimits _{N \rightarrow \infty } {\ln \tau \over N} \approx A_{\mathrm{out}}$$, with
    $$\begin{aligned} A_{\mathrm{out}} = \sum _{i=1}^{d_{\max }} P \left( d_{\mathrm{out}} = i \right) \ln \left( 1 + i D_{\mathrm{out}} \right) - {\gamma \over \kappa } D_{\mathrm{out}} \end{aligned}$$
    where $D_{\mathrm{out}}$ is the solution of
    $$\begin{aligned} {\kappa \over \gamma } \sum _{j=1}^{d_{\max }} {j P \left( d_{\mathrm{out}} = j \right) \over 1 + j D_{\mathrm{out}}} = 1 ; \end{aligned}$$
  (ii) for two populations with $\kappa ^{(1)} = \kappa ^{(2)}$, $\gamma ^{(1)} = \gamma ^{(2)}$,
    $$\begin{aligned} d^{(1)}_{\mathrm{out}} \le _{cv} d^{(2)}_{\mathrm{out}} \Rightarrow A_{\mathrm{out}}^{(1)} \ge A_{\mathrm{out}}^{(2)} ; \end{aligned}$$
    in particular, $A_{\mathrm{out}}$ is maximised when every individual has the same out-degree, $P \left( d_{\mathrm{out}} = \mu \right) = 1$.
- Suppose that $P \left( d_{\mathrm{out}} = \mu \right) = 1$, so every individual has the same out-degree, and that infectious periods follow an Erlang distribution. Then
  (i) $$\displaystyle \lim \nolimits _{N \rightarrow \infty } {\ln \tau \over N} \approx A_{\mathrm{in}}$$, with
    $$\begin{aligned} A_{\mathrm{in}} = \sum _{i=1}^{d_{\max }} P \left( d_{\mathrm{in}} = i \right) \ln \left( 1 + i D_{\mathrm{in}} \right) - {\gamma \over \kappa } D_{\mathrm{in}} \end{aligned}$$
    where $D_{\mathrm{in}}$ is the solution of
    $$\begin{aligned} {\kappa \over \gamma } \sum _{j=1}^{d_{\max }} {j P \left( d_{\mathrm{in}} = j \right) \over 1 + j D_{\mathrm{in}}} = 1 ; \end{aligned}$$
  (ii) for two populations with $\kappa ^{(1)} = \kappa ^{(2)}$, $s^{(1)} = s^{(2)}$, $\gamma ^{(1)} = \gamma ^{(2)}$,
    $$\begin{aligned} d^{(1)}_{\mathrm{in}} \le _{cv} d_{\mathrm{in}}^{(2)} \Rightarrow A^{(1)}_{\mathrm{in}} \ge A^{(2)}_{\mathrm{in}} ; \end{aligned}$$
    in particular, $A_{\mathrm{in}}$ is maximised when every individual has the same in-degree, $P \left( d_{\mathrm{in}} = \mu \right) = 1$.

Note that our asymptotic results (Theorem 1) are exact for the model with transition rates given by Table 1, but approximate under the annealed network interpretation.

A third open issue is to allow for more general infectious period distributions in the case of heterogeneous infectivity (or heterogeneous out-degree, under the network interpretation). We conjecture that Theorem 1(i) remains valid, and hence also Theorem 2(i) and Theorem 4(a), if infectious periods are allowed to follow an Erlang, rather than exponential, distribution. Indeed, Fig. 3 suggests that the mean persistence time $\tau$ is unchanged when ${{\varvec{\lambda }}} , {{\varvec{\mu }}}$ are interchanged. A difficulty here is that, in contrast to the case of heterogeneous susceptibilities, we have not been able to find complete solutions $U ( {\varvec{\theta }} ) , V ( {{\varvec{y}}} )$ to the relevant Hamilton–Jacobi equations even for the case of exponentially distributed infectious periods, but only, in proving Theorem 1(i), to evaluate $U ( {\varvec{\theta }} )$ along one particular trajectory.

One advantage of simple asymptotic formulae such as provided by Theorem 1, as opposed to the exact formula (3), is that they provide a route to qualitative results such as Theorem 2, that increasing heterogeneity reduces (at least to leading order) the expected persistence time of infection, and in particular that persistence time is maximised in a homogeneous population. Theorem 2 establishes this ordering when heterogeneity is in either infectivity or susceptibility; figure 2 suggests that the result remains true even when both types of heterogeneity are present. It is interesting to compare with the results contained in section 5 of Clancy and Pearce (2013) regarding the effect of such heterogeneities upon the (large population) mean endemic prevalence level $y^* = \sum _{i=1}^k y_i^*$. Theorem 7(i) and theorem 10 of Clancy and Pearce (2013) show, respectively, that heterogeneous infectivity alone has no effect upon the endemic prevalence level $y^*$, whereas heterogeneous susceptibility alone can only decrease $y^*$, with ${{\varvec{\mu }}}^{(1)} \prec _{{\varvec{f}}} {{\varvec{\mu }}}^{(2)} \Rightarrow y^{*(1)} \ge y^{*(2)}$, corresponding to our Theorem 2(ii) for persistence times. When both types of heterogeneity are combined, theorem 8 of Clancy and Pearce (2013) shows that if infectivity and susceptibility are non-negatively correlated ($\sum _{i=1}^k \lambda _i \mu _i f_i \ge 1$) then the endemic prevalence level cannot be greater than for a homogeneous population with the same $R_0$ value. However, theorem 9 of Clancy and Pearce (2013) together with numerical work shown in figure 3 of Clancy and Pearce (2013) demonstrates that when infectivity and susceptibility are negatively correlated it is possible for the endemic prevalence level to be greater than in the homogeneous case (with $R_0$ values matched). This presents an interesting contrast to our numerical results in Fig. 2, where heterogeneities were found to always decrease (to leading order) the expected persistence time, regardless of whether ${{\varvec{\lambda }}} , {{\varvec{\mu }}}$ are positively or negatively correlated.

There is a slightly counter-intuitive aspect to the above results, in that an increase in endemic prevalence level may correspond to a decrease in expected persistence time. This is easily resolved by observing that an increase in prevalence level may be accompanied by a corresponding increase in variability, leading to faster extinction of infection. The effect of heterogeneities upon the variability of the quasi-stationary distribution is studied in section 6 of Clancy and Pearce (2013), via an Ornstein–Uhlenbeck diffusion approximation that leads to a multivariate normal approximation to the quasi-stationary distribution ${\varvec{q}}$. The variability of this approximating normal distribution is then used as a proxy measure of persistence time. This approach seems reasonable in terms of qualitative comparisons between infection models, and is common in the literature. However, the approach is known to give a very bad numerical approximation to mean persistence time, with incorrect leading-order asymptotic behaviour, due to the failure in the lower tail of the normal approximation to the quasi-stationary distribution (Doering et al. 2005; Clancy and Tjia 2018). The methods of the current paper, in contrast, deal directly with the expected persistence time and yield correct leading-order asymptotic formulae.

## Appendix

The Hamilton–Jacobi equations (7) amount to two alternative ways of expressing the eigenvector equation (3), retaining only terms of leading order in *N* in the limit as $N \rightarrow \infty$. We briefly outline the derivation of each equation, referring the reader to Assaf and Meerson (2017) and references therein for full details.

For a process with transition rates of the form (4), Eq. (3) may be written as

30 $$\begin{aligned} \sum _{{{\varvec{l}}} \in L} \left( q_{{{\varvec{x}}}-{{\varvec{l}}}} W_{{{\varvec{l}}}} \left( {{{\varvec{x}}} - {{\varvec{l}}} \over N} \right) - q_{{{\varvec{x}}}} W_{{{\varvec{l}}}} \left( {{{\varvec{x}}} \over N} \right) \right) = - (\tau N)^{-1} q_{{{\varvec{x}}}} \quad \hbox { for } {{\varvec{x}}} \in C . \end{aligned}$$

Suppose (*ansatz*) that $q_{{{\varvec{x}}}} = \exp \left( - N V ( {{\varvec{x}}} / N ) + o(N) \right)$ for some function $V(\cdot )$. Writing ${{\varvec{y}}} = {{\varvec{x}}} / N$, then $V \left( {{\varvec{y}}} - {{{\varvec{l}}} \over N} \right) = V ( {{\varvec{y}}} ) - ( {{\varvec{l}}}^T / N ) {\partial V \over \partial {{\varvec{y}}}} + o (1/N)$ and hence

$$\begin{aligned} q_{{{\varvec{x}}} - {{\varvec{l}}}} = \left( \exp \left( {{\varvec{l}}}^T {\partial V \over \partial y} \right) + o(1) \right) q_{{{\varvec{x}}}} . \end{aligned}$$

Similarly, $W_{{{\varvec{l}}}} \left( {{\varvec{y}}} - {{{\varvec{l}}} \over N} \right) = W_{{{\varvec{l}}}} ( {{\varvec{y}}} ) + o(1/N)$. Retaining only terms of leading order in *N*, and assuming that $\tau$ is sufficiently large for the right hand side of Eq. (30) to be neglected, then Eq. (30) reduces to

$$\begin{aligned} \sum _{{{\varvec{l}}} \in L} W_{{{\varvec{l}}}} ( {{\varvec{y}}} )\left( \exp \left( {{\varvec{l}}}^T {\partial V \over \partial {{\varvec{y}}}} \right) - 1 \right) = 0 . \end{aligned}$$

That is, $$H \left( {{\varvec{y}}} , {\partial V \over \partial {{\varvec{y}}}} \right) = 0$$.

Next, consider the moment generating function $M ( {\varvec{\theta }} ) = \sum _{{{\varvec{x}}} \in C} {\mathrm{e}}^{{\varvec{\theta }}^T {{\varvec{x}}}} q_{{{\varvec{x}}}}$. Multiplying Eq. (30) by ${\mathrm{e}}^{{\varvec{\theta }}^T {{\varvec{x}}}}$ and summing over ${{\varvec{x}}}$ we find

31 $$\begin{aligned} \sum _{{{\varvec{x}}} \in C} \sum _{{{\varvec{l}}} \in L} {\mathrm{e}}^{{\varvec{\theta }}^T {{\varvec{x}}}} q_{{{\varvec{x}}}} W_{{{\varvec{l}}}} \left( { {{\varvec{x}}} \over N} \right) \left( {\mathrm{e}}^{{\varvec{\theta }}^T {{\varvec{l}}}} - 1 \right)= & {} - (\tau N)^{-1} M ( {\varvec{\theta }} ) . \nonumber \\ \hbox {That is,} \quad \sum _{{{\varvec{l}}} \in L} \left( {\mathrm{e}}^{{\varvec{\theta }}^T {{\varvec{l}}}} - 1 \right) W_{{{\varvec{l}}}} \left( {1 \over N} {\partial \over \partial {\varvec{\theta }}} \right) M ( {\varvec{\theta }} )= & {} - (\tau N)^{-1} M ( {\varvec{\theta }} ) . \end{aligned}$$

Suppose (*ansatz*) that $M ( {\varvec{\theta }} ) = \exp \left( N U ( {\varvec{\theta }} ) + o(N) \right)$ for some function $U(\cdot )$, so that $U(\cdot )$ gives the leading-order term in the cumulant generating function of the quasi-stationary distribution ${\varvec{q}}$. Retaining only terms of leading order in *N* and assuming that $\tau$ is sufficiently large for the right hand side of Eq. (31) to be neglected, then Eq. (31) reduces to

$$\begin{aligned} \sum _{{{\varvec{l}}} \in L} \left( {\mathrm{e}}^{{\varvec{\theta }}^T {{\varvec{l}}}} - 1 \right) W_{{{\varvec{l}}}} \left( {\partial U \over \partial {\varvec{\theta }}} \right) = 0 . \end{aligned}$$

That is, $$H \left( {\partial U \over \partial {\varvec{\theta }}} , {\varvec{\theta }} \right) = 0$$.

Note that the Hamilton–Jacobi equations (7) thus have boundary conditions $U ( \mathbf{0} ) = V ( {{\varvec{y}}}^* ) = 0$. However, we can add an arbitrary constant to any solution of either Eq. (7) and still have a solution, and any such additive constant will cancel out when we come to estimate $(\ln \tau ) / N$ using Eq. (8). We therefore ignore the boundary conditions and instead choose additive constants so as to keep our presented solutions for $U( {\varvec{\theta }} ) , V ( {{\varvec{y}}} )$ as simple as possible.
